# Accounting for soil respiration variability – Case study in a Mediterranean pine-dominated forest

**DOI:** 10.1038/s41598-020-58664-6

**Published:** 2020-02-04

**Authors:** Ottorino-Luca Pantani, Fabrizio Fioravanti, Federico M. Stefanini, Rossella Berni, Giacomo Certini

**Affiliations:** 1 Dipartimento di Scienze e Tecnologie Agrarie, Alimentari, Ambientali e Forestali (DAGRI), Università degli Studi di Firenze, Piazzale delle Cascine 28, 50144 Firenze, Italy; 2 Dipartimento di Statistica, Informatica, Applicazioni “Giuseppe Parenti”, Università degli Studi di Firenze, Viale Morgagni 59, 50134 Firenze, Italy

**Keywords:** Carbon cycle, Carbon cycle, Projection and prediction, Environmental monitoring

## Abstract

The number of spots to monitor to evaluate soil respiration (R_s_) is often chosen on an empirical or conventional basis. To obtain an insight into the necessary number of spots to account for R_s_ variability in a Mediterranean pine-dominated mixed forest, we measured R_s_ all year long on sixteen dates with a portable gas-analyser in 50 spots per date within an area 1/3 ha wide. Linear mixed-effects models with soil temperature and litter moisture as descriptors, were fitted to the collected data and then evaluated in a Monte Carlo simulation on a progressively decreasing number of spots to identify the minimum number required to estimate R_s_ with a given confidence interval. We found that monitoring less than 14 spots would have resulted in a 10% probability of not fitting the model, while monitoring 20 spots would have reduced the same probability to about 5% and was the best compromise between field efforts and quality of the results. A simple rainfall index functional to select sampling dates during the summer drought is proposed.

## Introduction

Soils are the main carbon (C) reservoir of terrestrial ecosystems and contain about twice as much C as the atmosphere^1^. As a consequence, small changes in soil respiration (R_s_) – the efflux of CO_2_ to the atmosphere resulting from biological activity in soil – may have important consequences on climate and in fact received much attention in recent years^2,3^. Gathering R_s_ measurements from different biomes around the world is essential for obtaining a reliable estimate of soil CO_2_ efflux on a global scale^3,4^.

Reliable R_s_ data can only arise from direct measurement in the field, which still presents many challenges despite the fact that it has been carried out for more than a century. The early measurements of R_s_ were based on chemical absorption of CO_2_ within a closed chamber by alkali solutions and successive titration. Nowadays, an infrared gas analyser (IRGA) which measures the CO_2_ concentration build-up inside a dynamic chamber is generally used. Although the IRGA system is not free from artefacts and biases – which can however be minimized^3,5^ – it has the great advantage of allowing relatively fast measurements and, therefore, of measuring R_s_ in many different spots. Soil is actually a highly variable environment in terms of each of its features^6–8^. Features related to the soil biota are expected to be even more variable than the others^9^, both seasonally and spatially.

There is an extensive number of studies which have measured R_s_ in many ecosystem types (see the updated global dataset by Bond-Lambert and Thomson^10^); however, very few of them were concerned about how many spots should be measured in that specific environment to properly account for the intrinsic spatial variability of soil. One of these is the experiment set up by Saiz *et al*.^11^, who in a first rotation Sitka spruce chronosequence composed of four age classes in Ireland, first assessed that coefficients of variation in R_s_ varied largely during the year – being lowest during periods with highest R_s_ – then determined that on average the sampling strategy of 30 sampling spots per stand (of unspecified area) was adequate to obtain a R_s_ within 20% of its actual value with p = 0.05. Measuring R_s_ on a regular grid covering an area of 2400 m^2^ in a mature plantation of *Cryptomeria japonica* in Japan, Lee and Koizumi^12^ assessed that the spots required to produce a sample mean within ±10% of the full-population mean (p = 0.05) on three sampling dates were 75, 48, and 110. By random measurements, Rochette *et al*.^13^ showed that in a wheat crop the number of spots needed to estimate the R_s_ on one single hectare within 10% of its mean value decreased from 190 at the time of seeding to 30 at the end of the growing season.

Rodeghiero and Cescatti^14^ evaluated a method based on initial measurements in a number of randomly selected spots; this number was later reduced by a stratified sampling to the minimum required to adequately estimate R_s_. In practice, the spots were sorted according to their average R_s_ in the first three sampling dates and then equally divided into as many “strata” as the number of spots the researchers actually wanted to continue working on (to be selected randomly one per group). To evaluate the effectiveness of the random and stratified samplings, the authors re-sampled the experimental dataset with a Monte Carlo (MC) approach, varying the sub-sample size at each site. The stratified sampling was found to be effective only in those two ecosystems (of the three studied), where the temporal correlation of the fluxes was high. A similar approach has been used by Knohl *et al*.^15^, who measured R_s_ every 2 to 6 weeks for more than two years in a temperate mixed deciduous forest in Germany. Starting from several measurement sessions during the first year on 40–50 spots, aimed at capturing spatial variability in an area with unknown extension, the authors estimated that at least 8 spots were required to stay within ±20% of the expected mean with p = 0.05 and at least 44 to stay within ±10% of the same mean.

The fact is that every type of stratification inevitably causes some loss of information. Moreover, such an approach bases on the weak assumption that the spots chosen at the beginning of the experiment are constantly ranked in the same way in terms of R_s_. From papers dealing with R_s_ determined by the IRGA device and reporting enough information to infer the sampling density, we drew up Supplementary Tab. 1. The listed works were carried out all around the world, in various environments experiencing diverse types of climates and undergoing different land uses (mostly forests). The majority of these works were observational, *i.e*., performed without altering the natural conditions, while a few ones were designed, *i.e*., performed by modifying some climatic or physical features to account for the effects of plausible environmental changes. The spots were distributed randomly or regularly, along a line (transect-based) or on a grid (grid-based) on areas ranging from few square meters to 65 ha. Sampling density ranged from 1.29 to 12,727 spots per hectare (ha), where values over 300 spots ha^−1^ resulted from monitored surfaces which were objectively too small (less than 0.1 ha) to adequately capture the R_s_ spatial variation. Physical and biological controlling factors of CO_2_ efflux may in fact be different at larger scales. In most of the papers listed in Supplementary Tab. 1 the choice of the sampling density appears to be random or not adequately explained.

There are several papers dealing with R_s_ assessment in forests growing in areas with a Mediterranean climate, where weather seasonality and the spatial variability of vegetation structure are generally higher than in other environments^16–19^; nevertheless, few of them paid adequate attention to soil variability and the necessity of working on a number of spots sufficient to obtain a reliable estimate of R_s_.

The aim of this work is to get insight into the uncertainty in R_s_ assessment due to both the number of monitored spots and the seasonal variation in a soil under a pine-dominated forest experiencing Mediterranean climate. The data gathered was thus used to answer to the question: how many spots should be measured to capture R_s_ variability in this environment? For this purpose, soil temperature, soil and litter moisture, and R_s_ were measured on 16 dates throughout one year with a portable gas-analyser at 50 spots per day within an area 1/3 ha wide, assuming that the above figures would yield a labour-expensive yet reasonable oversampling. Some linear mixed effects models describing the dependence of R_s_ on soil temperature and moisture were fitted to the collected dataset and a MC simulation was run for each model to check the effect of a progressive reduction of sampling density on model fitting and the associated uncertainty. A simple index based on cumulative rain was used to select beforehand the most convenient dates of sampling during summer droughts. The ultimate aim of this study is optimizing the allocation of efforts without missing the core of information of the experiment.

## Results

**Figure 1:**
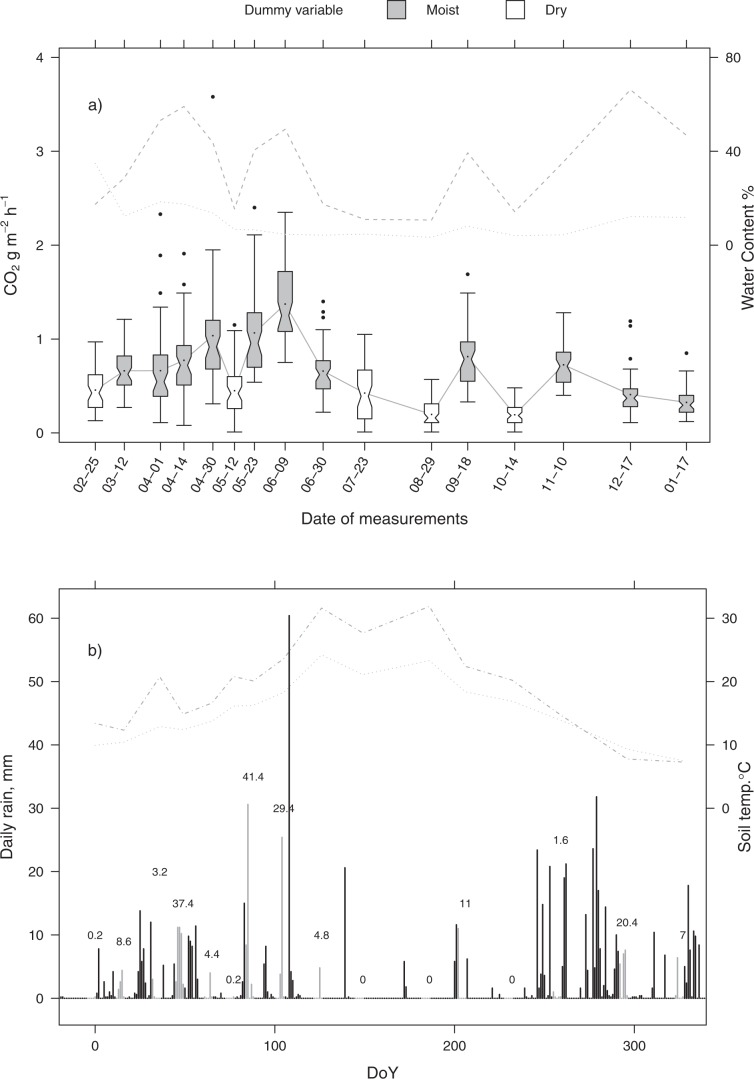
Top panel a) shows the CO_2_ fluxes measured at 16 dates (indicated as Julian days of 2008 on x axis) in the pine-dominated forest. Notches and dots in the boxplots are the median and the mean, respectively. The gray and white bodies indicate Moist and Dry conditions, respectively. The solid line connects the mean R_s_, while the dashed and dotted lines above the boxplots indicate litter and soil moisture (% on wet weight), respectively. Bottom panel b) shows the daily precipitations (bars), the mean temperature of soil (dotted line) and air (dotted-dashed line). The gray bars highlight the 4 days before the CO_2_ measurements while numbers indicate the cumulative precipitation (mm).

The temporal distribution of R_s_ values is shown in Fig. 1a, along with climatic data (Fig. 1b) and litter and soil moisture. Soil respiration was highly variable during the year and between the spots (Supplementary Tab. 1), ranging from a minimum of 0.01 g CO_2_ m^−2^ h^−1^ – measured in May, July, October, and August, when the highest recorded values were instead 1.15. 1.05. 0.57 and 0.48 g CO_2_ m^−2^ h^−1^, respectively – to a maximum of 3.58 g CO_2_ m^−2^ h^−1^ in April, when the lowest recorded value was 0.31 g CO_2_ m^−2^ h^−1^ (Supplementary Tab. 2). The time trend of R_s_ reflected that of litter moisture (solid and dashed lines in Fig. 1a, respectively), which supports the strong dependence of the first variable upon the second one. Only in December R_s_ did not rise with litter moisture, most probably because of temperatures being too low. At each date, the variability between the spots was high, with standard deviations ranging from 0.11 to 0.56 g CO_2_ m^–2^ h^–1^ (Supplementary Tab. 2). Boxplots in Fig. 1a show that R_s_ variability increased with increasing R_s_

### Re-sampling strategies and Monte Carlo simulations

**Figure 2:**
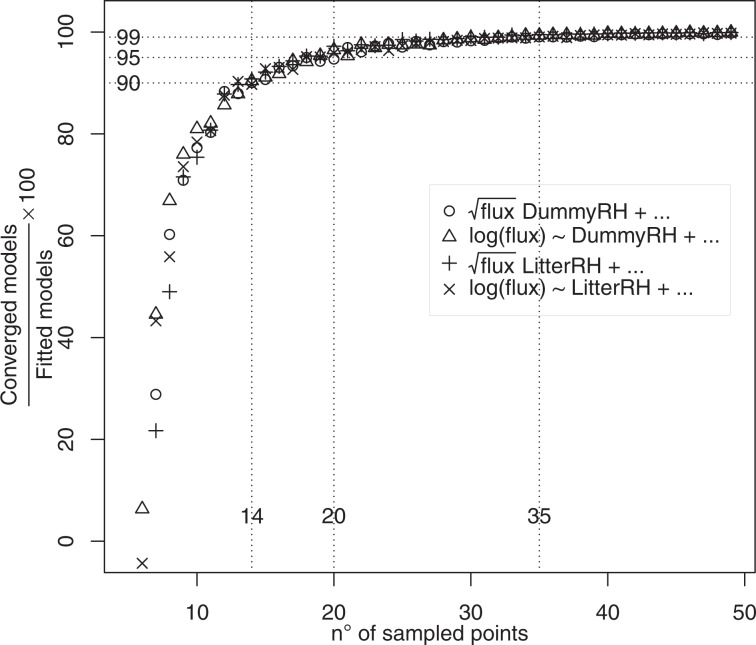
The ratio of converged models/fitted models (1001 converged models obtained) increases exponentially with the number of sampled spots. Vertical dotted lines are drawn at number of spots to monitor in order to have a 90, 95, and 99 ratio of converged/fitted models. RH = Relative Humidity, Moisture in the text.

The relationship between the number of spots (hereafter *MCspots*) – *i.e*., 49, 48, … 6, 5 – and the converged/fitted model ratio is shown in Fig. 2. All models displayed similar behaviour as the *MCspots* decreased. One important outcome is that it is necessary to monitor at least 14 spots to sufficiently account for R_s_ variability; in fact, sampling fewer spots would imply that more than 10% of the attempted models fails to converge and this percentage would rise exponentially as the number of *MCspots* decreases. On the contrary, for sample sizes greater than 20 and 35 *MCspots*, the percentage of failed models would drop below 5% and 1%, respectively.

**Figure 3:**
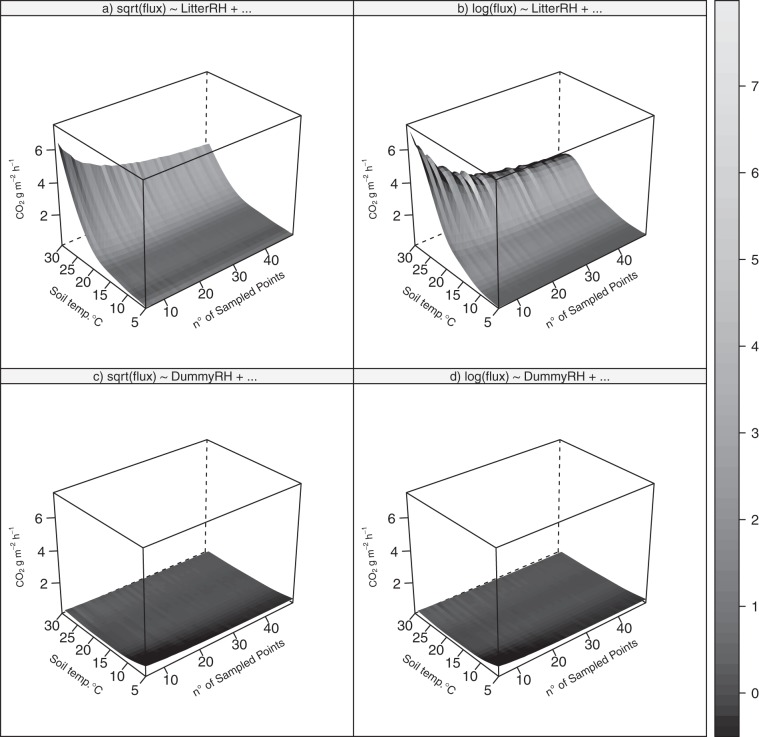
The uncertainty in prediction (l2-l1) calculated for four models.

**Figure 4:**
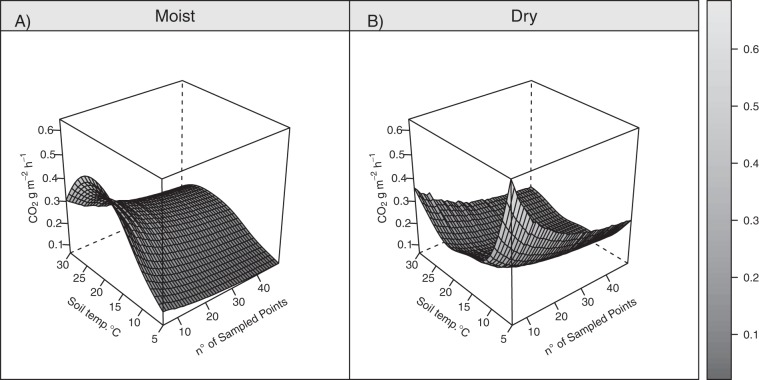
The uncertainty in prediction (l2-l1) calculated by model [1]. Two surfaces are reported, as the model has a binary dummy variable “Moisture” with Moist and Dry levels. The greater prediction intervals under Dry conditions is a direct result of both the Mediterranean climate and of the fact that the spots, when monitored, were described by Moist more often than by Dry conditions.

**Figure 5:**
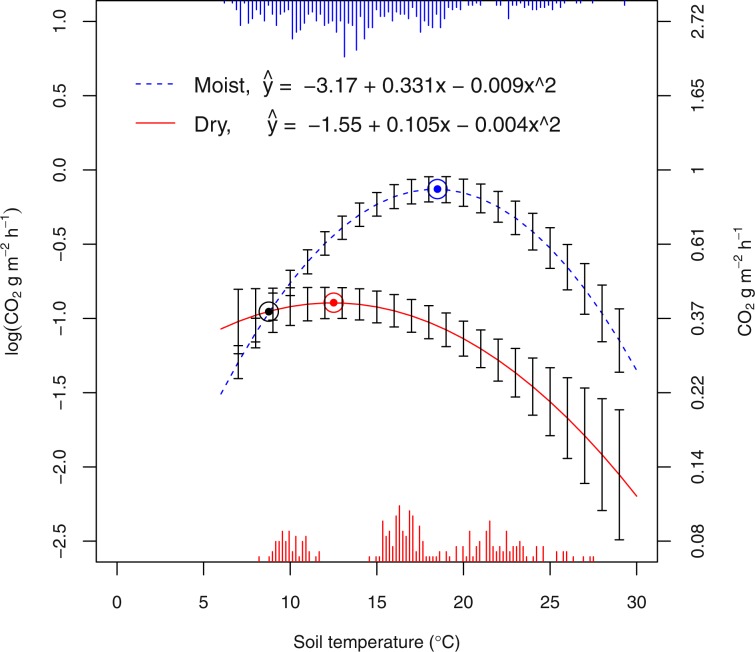
The fixed part of the mixed-effects model [1] described in the text. Equations were calculated from values reported in Table 2. The error bars (l2-l1, see text) cover the minimum (6 °C) and the maximum (29 °C) soil temperatures recorded in the field. The distribution of the temperature is shown as bars and split between Dry and Moist dates (bottom and top, respectively).

The surfaces shown in Fig. 3 are the result of uncertainty in prediction (z axis, CO_2_ flux in original units, g m^−2^ h^−1^), based on the four models we tested as a function of both *MCspots* (x axis) and soil temperature (y axis). The models with the dummy variable show much lower uncertainty in prediction. This favourable feature was not evident either from the BIC values (Supplementary Tab. 3) or the shape of quantile-quantile plots (Supplementary Fig. 1). Since the models with dummy variables – log- or square root-transformed R_s_ – gave quite similar results both in BIC and quality of residuals, the log-transformed response is here used for the discussion. Due to the presence of the dummy variable, it is possible to show the confidence interval for the two conditions, *Moist* and *Dry* (Figs. 4 and 5). The lowest and highest uncertainties were 0.06 and 0.64 CO_2_ g m^−2^ h^−1^ and occurred for *Moist* conditions at 5 °C for a sample size of 49, and for *Dry* conditions at 5 °C for a sample size of 5 spots, respectively. The surface for *Dry* conditions rises steeply in the corner with lower sample sizes and temperatures. While the effect of sample size is obvious, the effect of temperature may be due to the fewer measurement sessions under the *Dry* conditions (5 sessions vs. 11). Soil respiration peaked at 18 °C and 12 °C for *Moist* and *Dry* conditions (Fig. 5), where it amounted to 0.86 and 0.40 CO_2_ g m^−2^ h^−1^, respectively. The fluxes at the two moisture conditions were the same at about 9 °C, where both amounted to 0.38 CO_2_ g m^−2^ h^−1^.

## Discussion

### Comparison of Values

The mean R_s_ we found lies on the higher side of the range of values shown in Supplementary Tab. 1 (min. 0.06, 1^st^ quartile 0.26, median 0.43, mean 0.43, 3^rd^ quartile 0.6, max 0. 99 g CO_2_ m^−2^ h^−1^). As regards the Mediterranean ecosystems specifically, Emran *et al*.^20^ under *Pinus pinea* measured an annual mean R_s_ of 0.69 ± 0.47 g CO_2_ m^−2^ h^−1^, while in two plots of a Scots pine-dominated mixed forest, Barba *et al*.^21^ measured an R_s_ of 0.44 g CO_2_ m^−2^ h^−1^ (0.04 CV, n = 97) and 0.35 (0.03 CV, n = 98) while Rey *et al*.^17^ reported a mean annual R_s_ of 0.46 g CO_2_ m^−2^ h^−1^ for a coppice of *Quercus cerris* L. The Mediterranean climate is characterized by the alternation of a growing wet season and a non-growing dry season, which clearly have different mean seasonal R_s_^22^. As a consequence, every mean annual R_s_ to be meaningful should rely on as many as possible sampling campaigns distributed on the basis of the respective lengths of the two seasons. The mean R_s_ we found is thus perfectly aligned with the ones cited above. Additionally, the R_s_ range of values we measured is comparable with those found by Rey *et al*.^17^ (0.19 to 1.01 g CO_2_ m^−2^ h^−1^) and Joffre *et al*.^18^ and Asensio *et al*.^23^ in a *Quercus ilex* forest (0.06 to 0.80 g CO_2_ m^−2^ h^−1^ and 0.13 to 0.54 g CO_2_ m^−2^ h^−1^, respectively).

Land use is another factor of R_s_ variability in Mediterranean areas. In this regard, Oyonarte *et al*.^24^ in six different land uses (forest and agricultural sites) found values ranging from 0.06 g CO_2_ m^−2^ h^−1^ in the dry period (summer) to 0.28 g CO_2_ m^−2^ h^−1^ in the growing season (spring), while in a sparse *Pinus halepensis* forest, an olive grove, and an abandoned field, Almagro *et al*.^19^ found mean R_s_ of 0.33, 0.27, and 0.18 g CO_2_ m^−2^ h^−1^, respectively. Soils under pine woods, as the one we investigated, thus seem to release more CO_2_ than soils with different vegetation cover^19,20^.

### Considerations on sampling density and allocation of resources

Supplementary Tab. 1 collates some relevant data from 38 papers including this one. The sampling densities range from 1.29 to 12,727 spots ha^−1^. Two aspects must be considered: the first one is that nine densities are above 288 spots ha^−1^ and result from designed experiments that involved areas from 0.04 to 0.006 ha. Working on so small areas of course does not allow reliably evaluating R_s_ variability at ecosystem level; on the other hand, such sampling densities if exported on rather large surfaces are impossible to manage. To achieve a higher density, some monitoring plans were based on the use of two or more IRGA devices or two or more days to collect data^21,25,26^; nevertheless, also these plans consisted of about 50 spots per day. Tedeschi *et al*.^27^ is the only exception with 100 spots per day per device, but these authors reduced the number of spots to 20 right after a first screening. A single operator can not monitor much more than 50 spots per day, therefore he must size the area to monitor on such a basis. Should the sampling campaign base on two or more devices, the instrumental error of the devices must be minimal and as similar as possible in order to work together. Furthermore, in the case of the Mediterranean climate, spreading the measurement session over two days can imply dramatic changes in weather and, thus, in R_s_ values, especially depending upon rains (see next section).

### Considerations on temperature and moisture

As expected, temperature was a driving factor of R_s_, to a varying extent depending upon *Moist* and *Dry* conditions (Table 1 and Figs. 2 and 5). The models describing the dependence of R_s_ on temperature are generally based on the Arrhenius equation, so exponential functions frequently occur in the literature^19,28–31^. Although we did not find significant differences between the log and square root transformations in our experiment, we used the log transformation because of its wider acceptance in the literature. Nevertheless, we used a parabolic equation on the right side of the models, since all our attempts to build a model in exponential form were unsatisfactory due to the bad shape of residuals and the poor significance of the model parameters. The purpose of this study was to find the relationship between R_s_ uncertainty and the number of sampled spots; hence, the building of the model was data-driven.

**Table 1:** Estimated coefficients, standard errors, t-test, and p-values of soil respiration model based on soil temperature and litter moisture as a binary dummy variable. Graphical details of the model are reported in Supplementary Figs. 1, 3 and 4.

	Est. Coef.	Std. Error	DF	t-value	p-value
Moist conditions	−3.1712	0.1335	726	−23.739	<10^−3^
Dry conditions	1.6214	0.3490	726	4.645	<10^−3^
Soil temperature, °C	0.3312	0.0161	726	20.481	<10^−3^
(Soil temperature, °C)^2^	−0.0090	0.0004	726	−18.070	<10^−3^
Dry conditions*Soil temperature, °C	−0.2260	0.0452	726	−4.998	<10^−3^
Dry conditions*(Soil temperature, °C)^2^	0.00479	0.0013	726	3.438	<10^−3^

BIC: 1029.74; logLik −431.615.

The dependence of R_s_ on litter and soil moisture was evident at our site, just as in other Mediterranean ecosystems^17,32,33^. In particular, rain pulses were able to drastically increase R_s_ in the dry season (June 9^th^ and September 18^th^, see Fig. 1a). The infrequent rains in summer in Mediterranean climates have a substantial impact on R_s_^32^, so much to represent actual “hot moments” throughout the year^34^. Almagro *et al*.^19^ meaningfully proposed as a proxy for the effect of rain pulses on R_s_ the “rewetting index”, *RWI* = *P/t*, where *P* is the mm of precipitation and *t* the days passed since the previous precipitation. Such a proxy actually accounted for R_s_ during the long lasting summer drought better than soil moisture. We based our model on an alternative proxy, the cumulative precipitation exceeding 0.5 mm of the four previous days (*cp*): nonetheless, we cannot exclude that in regions with different climatic conditions, other timespans may work better.

Using indexes based on climate rather than physical measurements of soil moisture, allows for better comparison between studies, since soil moisture measurements are little comparable. In fact, the various studies conduct soil moisture measurements using different 1) methods (from gravimetric to volumetric, discrete vs continuous), 2) control sections, and 3) kinds of probes/sensors, which were often not calibrated to the local conditions. Another practical advantage of a climate index is that it can be calculated a priori and off-site. While soil/litter moisture must be measured each time in the field, *cp* can be easily monitored remotely and when it exceeds a critical value going to perform CO_2_ measurements.

### Considerations on alternative models

Using a model substantially reduces the number of spots to base on for measuring R_s_. In fact we calculated, for each sampling date, the number of spots to monitor according to the formula proposed by Petersen and Calvin^35^ and used by several authors^14,21,36,37^

$${\rm{n}}={{\rm{t}}}^{2}\ast {{\rm{s}}}^{2}/{{\rm{D}}}^{2}$$

where n is the sample size, t is the *t*-statistic (two-way test) for a given confidence level and degrees of freedom (95% in the present case), s is the standard deviation of the full population (50 spots per date in this case), and D is the specified error limit, i.e. the width of the desired interval around the full population mean in which a smaller sample mean is expected to fall^35^.

The results for 1/3 ha are shown in Table 2 and are much higher than those estimated by using model 1. From a predictive point of view there is no difference between using a model that considers only the mean^35^ and a model that considers more variables. On the other hand, the minor effort of recording variables such as soil temperature and moisture is worthwhile, since it dramatically reduces the number of spots to monitor in terms of CO_2_.

**Table 2:** Number of spots to monitor in 1/3 ha to restrain the variability within an uncertainty of 10% and 20% of the estimated mean value, according to the formula reported in Petersen and Calvin^35^.

Date	Coef. of variation 5%	Coef. of variation 10%
25/02/2008	369	92
12/03/2008	164	41
01/04/2008	654	164
14/04/2008	334	83
30/04/2008	468	117
12/05/2008	459	115
23/05/2008	273	68
09/06/2008	152	38
30/06/2008	270	68
23/07/2008	880	220
29/08/2008	792	198
18/09/2008	237	59
14/10/2008	541	135
10/11/2008	147	37
17/12/2008	424	106
17/01/2009	334	83

## Conclusion

The methodology here described – data oversampling, model building, Monte Carlo simulations – allowed reaching the principal goal of our work, *i.e*., to determine the number of spots necessary to capture R_s_ variability. This number turned out to not be less than 14 in 1/3 ha. Sampling more spots improves the precision of estimation, and 20 spots appeared to be the best compromise between field efforts and the quality of the result. In fact, in this case the probability of not finding a model dropped to less than 5%, while adding further spots does not bring any substantial benefit in terms of converged/fitted models ratio.

Another conclusion is that during the hot dry summer, a simple index based on cumulative precipitation can be used to establish the best dates to detect important CO_2_ pulses.

Overall, our findings encourage a more rational allocation of resources in both time and space for those aimed at measuring soil respiration in similar environments.

## Materials and Methods

### Study area

The study area, Pianacci (43°44′31.81″N, 11°06′14.76″E), is a forest stand located about 10 km south of Florence, Italy. The stand is dominated by maritime pine (*Pinus pinaster* Aiton) and, in suborder, Italian cypress (*Cupressus sempervirens* L.), with manna ash (*Fraxinus ornus* L.) and holm oak (*Quercus ilex* L.) as ancillary species (Supplementary Fig. 2). The climate is typically Mediterranean, with warm and dry summers and relatively cold and wet winters. Data from a weather station 3.5 km away from the study area and referring to the period 1994–2008 accounted for a mean annual precipitation of 764.3 mm, with November as rainiest month (119 mm) and July as the driest (24.4 mm), and a mean annual temperature of 14.8 °C, with January as coldest month (mean 6.5 °C) and July as the warmest (mean 24.2 °C). The terrain is located 216 to 224 m a.s.l. and has a mean slope of 5% and a west to southwest aspect. The soil formed on Oligocene sandstone chiefly composed of quartz, feldspars, and phyllosilicates and somewhere intercalated with thin siltstone layers comprising calcite, quartz, plagioclases, and phyllosilicates. It is a *Brunic Arenosol* of the World Reference Base for Soil Resources^38^ and shows an O-A-Bw-BC-C sequence of horizons. Some basic characteristics determined in a soil profile opened approximately in the centre of the stand are shown in Table 3. The very low occurrence of rock fragments in the topsoil (A and B horizons) reveals past agricultural land use.

**Table 3:** Some basic characteristics of the soil at the study area.

Horizon	Depth	Rock fragments	Sand	Silt	Clay	C	N	pH_H2O_	pH_KCl_
	cm	% weight	g kg^−1^	g kg^−1^	g kg^−1^	%	%		
O	2–0	2.0	na	na	na	11.66	0.49	5.15	4.33
A	0–10	2.6	816	181	3	0.84	0.02	5.97	4.28
B	10–24	1.8	873	112	15	0.58	nd	6.09	4.04
BC	24–39	20.5	878	111	11	0.43	nd	6.21	4.05
C	39+	65.7	467	428	105	0.30	nd	6.38	4.33

na = not applicable, nd = not detectable.

### Monitoring strategy and R_s_ measurements

Soil respiration was measured from February 2008 to January 2009, monthly except in April-May-June – the period of highest biological activity – when four extra measurement sessions were carried out (Fig. 1a). The CO_2_ efflux from soil was determined by an EGM-1 PP Systems portable gas analyser (Hitchin, UK) coupled with an SRC-1 closed air-circulation chamber 1.17 dm^3^ in volume. Within a 1/3 ha wide area we selected 50 spots to monitor throughout the year by a randomization procedure, excluding the outermost four-meter-wide strip of the stand to avoid any border effect. Based on the data of Supplementary Tab. 1 such a sampling density was assumed to be high enough to capture most R_s_ variability and to perform a Bootstrap resampling procedure aimed at finding the minimum number of spots necessary to estimate R_s_ with an uncertainty close to 10% of the mean of the population.

A stake was driven into the soil 10 cm north of each spot to localize it. We did not place permanent collars into the soil to prevent lateral gas leakage during measurement, as these have been shown to lead to greater underestimation of R_s_ due to their severing fine roots and the hyphae of mycorrhizal fungi and, possibly, modifying soil temperatures^39–41^. The R_s_ measurements were thus carried out by gently inserting the rimmed edge of the chamber 1 cm into the mineral soil and holding the chamber steady during the measurement, to virtually avoid uncontrolled exchanges of air to and from the chamber. At each spot, the temperature of both the atmosphere at ground level and the soil at a depth of 10 cm were recorded. All measurement sessions started at about 11:00, proceeding non-stop in an ordered sequence from spot no. 1 to spot no. 50, which was approximately measured at about 16:00; hence, spending on average about 6 minutes per spot. At each measurement session, two composite samples of both litter layer (O horizon) and top mineral soil were assembled from throughout the area to gravimetrically determine moisture by oven drying at 105 °C to constant mass.

### Statistical analysis and model building

In the analysis of data, the CO_2_ flux (Y, response variable) was considered: 1) on the original scale; 2) after log transformation; 3) after square root transformation. Explanatory variables were: soil temperature, litter and soil moisture (water % on wet weight). Moisture variables were considered: (a) on the original quantitative scale and (b) after transformation into a binary dummy variable (more details here below). Fitting and examination of linear mixed-effect models were performed following Pinheiro and Bates^42,43^. In particular, within each model as in 1), 2), and 3) above-cited points, selection of linear predictors for fixed effects was performed according to BIC (Bayesian Information Criterion) values^44^. In the case of litter moisture, fitted models also took into consideration the above-cited (i) and (ii) alternatives about moisture. Variance heteroscedasticity was considered by introducing random effects associated to sampling dates in all models.

For each best model on the chosen scale of the response (labels 1, 2, 3, above) the analysis of residuals^45^ was done to look for possible evidences of violations in model assumptions. The best models resulting from the above steps were then exploited in the MC simulation.

Litter moisture was found to be a better predictor than mineral soil moisture (Supplementary Tab. 4) and no further improvements of the model came either from the insertion of a cubic parameter or the elimination of the quadratic term (Supplementary Tab. 3).

Whatever the scale for the response variable, the final model matrix X after selecting the best model refers to the following fixed effects: (i) *Moisture*, defined as in labels (a) and (b) above; (ii) *Soil Temperature* and its square; (iii) the first order interaction term: *Moisture* * *Soil Temperature*, and (iv) Moisture * Soil Temperature^2^.

The matrix Z for random effects has the following hierarchical structure: (i) Sampled Spot and (ii) Soil Temperature within Sampled Spot.

Thus, the expected value of the response is:

1 $$\begin{array}{c}E[Y|X,Z]\sim Moisture+Soil\,Temperature+Soil\,Temperatur{e}^{2}\\ +\,Moisture\ast Soil\,Temperature+Moisture\ast Soil\,Temperatur{e}^{2}\end{array}$$

The random part of the model defines an intercept at a given date, which is randomly shifted from the value taken in the fixed part of the model. Random fluctuations of the coefficient for soil temperature are also introduced within the sampling date. Such random effects are normally distributed with null expectation. A variance parameter was introduced at each sampling date, so that the variance-covariance matrix of residuals was diagonal but not constant.

Models for transformed responses (labels 1, 2, 3) were adopted with the aim of checking the assumption of normality. A binary dummy variable (label a) was obtained by the sum of the daily precipitations of four days before the sampling date (*cp*, cumulative precipitation); hence, if *cp* was less than 0.5 mm, the dummy variable was set to *Dry*, otherwise it was set to *Moist*. The “dry” dates were identified at first on an empirical basis, *i.e*., as those where low values of both litter moisture and R_s_ were observed, namely 02–25, 05–12, 07–23, 8–29, and 10–14 (Fig. 1a). Sampling dates were those in which the cumulative precipitation was lower than 0.5 mm in the four previous days. Of course, using values other than 0.5 mm and 4 days, different “dry” dates resulted.

The combination of the CO_2_ flux (as such, log or square-root transformed) with the two variables (litter moisture or the dummy variable) provided six candidate models for MC simulation (Supplementary Tab. 5 and Fig. 1). The analysis of residuals showed evidence of violation in the assumptions in models with non-transformed CO_2_ flux as response (Supplementary Fig. 1) and did not indicate any superiority of one model over the others. Therefore, only the four models with log- or square root-transformed underwent Bootstrap re-sampling and MC simulations.

### Uncertainty associated with monitoring fewer spots

The uncertainty associated with monitoring fewer spots was evaluated by Bootstrap resampling^46,47^. Several Bootstrap runs were performed by progressively decreasing the number of spots (*MCspots*) down to 5: that is 49, 48, …6, 5. In each Bootstrap run, for a given *MCspots* value a random sample with replacement was drawn from the complete dataset (thus forming a Bootstrap Sample dataset, *BSdataset*); a model was fitted on this *BSdataset* and, if model fitting converged, confidence intervals were recorded. The stopping rule for resampling was 1001 models successfully fitted to Bootstrap samples (without errors due to convergence, aliasing, etc.).

Two main consequences were expected from the reduction of the *MCspots* value: (i) the failure of convergence during model fitting and (ii) the increase of uncertainty of parameter estimates, as captured by the difference between the two endpoints of the confidence interval (95%). The R software^48^ and its libraries, nlme^49^ and lattice^50^, were used for data entry, model fitting, and MC simulations.

### Data availability

The dataset is available at https://doi.pangaea.de/10.1594/PANGAEA.896345

## Supplementary information

Supplementary information .
