# Supplementary material for: Accounting for soil respiration variability – Case study in a Mediterranean pine-dominated forest
Source: Sci Rep. 2020 Feb 4;10:1787. doi: 10.1038/s41598-020-58664-6 (PMC7000697; doi:10.1038/s41598-020-58664-6)
Supplement: Supplementary file 1 — Supplementary information . [file 41598_2020_58664_MOESM1_ESM.pdf]

Supplementary information for

Accounting for soil respiration variability – Case study in a Mediterranean pine-dominated forest

Ottorino-Luca Pantani*[1], Fabrizio Fioravanti[1], Federico M. Stefanini[2], Rossella Berni[2], Giacomo Certini[1]

Affiliations:

[1] Dipartimento di Scienze e Tecnologie Agrarie, Alimentari, Ambientali e Forestali (DAGRI), Università degli Studi di Firenze, P.le delle Cascine 28 – 50144 Firenze, Italy

[2] Dipartimento di Statistica, Informatica, Applicazioni "Giuseppe Parenti", Università degli Studi di Firenze, Viale Morgagni 59 – 50134 Firenze, Italy

Table S1. Sampling densities and other materials and methods in papers dealing with soil respiration determined by a portable analyser Rows are sorted by increasing sampling density, spots per ha. The unit *spots per ha* was calculated to standardize the high variability in sampling size and surface, thus allowing direct comparison of data.

**Type of study**	**Land use**	**Location**	**Climate**	**Sampling design**	**Sampling frequency and duration**	**Daily frequence and hour**	**Year(s) of the experiment**	**Reference**	**Method of measurement**	**Mean R_s (g CO_2 m^{-2} h^{-1})**	**Monitored surface (ha)***	**Numbers of spots measured**	**Sampling density (spots per hectare)**
Observational	Forest	France	Temperate	Transect-based	Every two–three weeks, over 18 months	Between 7:30 and 16:00	2003-2004	[1]	Closed dynamic chamber LI-COR 6252	0.26	65	84	1.29
Observational	Forest. grove. pasture. shrubland	Spain	Mediterranean montane	Random	13 sessions	Once a day. between 13:00 and 15:00	Autumn 2008 to autumn 2009	[2]	Both MultiRAE IRGA Monitor PGM54. RAE Systems Inc.. and soda lime	0.69	0.5	3	6

Observational	Forest	California, USA	Mediterranean	Random	Every two weeks	From 9:00 to 17:00	From June 2011 to December 2012	3	Closed dynamic chamber LI-COR 6000 / 6400	Not calculable	3	29	9.6
Observational	Forest	Spain	Mediterranean	Random	Monthly	9:00 – 12:00	January 2006 and December 2007	4	Closed dynamic chamber LI-COR 6400-09	0.37	1.8	30	16.7
Observational	Olive grove	Spain	Mediterranean	Random	Monthly	9:00 – 12:00	January 2006 and December 2007	4	Closed dynamic chamber LI-COR 6400-09	0.35	1.2	24	20
Observational	Savannah	South Africa	Semi-arid	Transect-based	Twice per month		From November 2004 to July 2006	5	Closed dynamic chamber EGM-2 PP-Systems	0.99	0.785	16	20
Observational	Forest	Malaysia	Tropical. dry	Grid-based	2 times		March 2000	6	Closed dynamic chamber LI-COR 6400**	0.63	2	50	25

Observational	Forest	Thailand	Tropical. dry	Grid-based	2 times		February and September 2005	7	Closed dynamic chamber LI-COR 6400**	0.72	2	50	25
Observational	Forest	Italy	Alpine	Random	Irregularly, for a total of 14 sampling dates		Spring 2000 to spring 2002	8	Closed dynamic chamber LI-COR 6400	Not calculable	0.66	21	32
Observational	Forest and grassland	Belgium	Temperate maritime	Random	Once a month Two plots (Scots pines without understory and pedunculate oaks without understory) of ten were more intensively monitored (66 measure		From early January 2001 until the end of December 2001	9	Closed dynamic chamber SRC-1. PP-Systems	Not calculable	0.3 to 1.9 (nine plots)	10	33 to 5

					ment dates)								
Observational/ designed	Desert shrub	China	Continen tal arid	Rando m	Once per month	Every 2 h since 8:00 to 20:00	May 2005 to Octobe r 2006	[10]	Closed dynamic chamber LI-COR 8100	0.09	0.09	5	56
Designed	Cold desert	USA. Utah	Cold-desert	Transe ct-based	Once per month for two years		From Februar y to Novem ber in 2002 and 2003	[11]	Closed dynamic chamber LI-COR 6400-09	0.06	0.44	25	57
Observational	Forest	Germany	Tempera te montane	Rando m	Once per day		From April to Decem ber 1999	[12]	Open dynamic soil chamber IRGA	Not calcul able	0.25	15	60
Observational	Mature tree plantat ion	Japan	Asian monsoon	Grid-based	Three dates: in July, Septemb er, and Novemb er	From 11:00 to 13:00	2005		Closed dynamic chamber LI-COR 6400-09	0.36	0.24	15	62.5
Observational	Forest	Germany	Subocea nic	Grid-based	Two measure ment	During the daytime	July and Decem	[13]	Closed dynamic chamber	0.49	0.5	36	72

			subcontinental		campaigns	of one or two days each campaign.	ber 2000		LI-COR 6400-09				
Observational	Grassland	Italy	Alpine	Random	Irregularly, for a total of 14 sampling dates		October 2002 and July 2003	[8]	Closed dynamic chamber LI-COR 6400	Not calculable	0.24	20	83
Observational	Forest	North eastern China	Continental monsoon	Grid-based	Three sampling dates, avoiding rain	10:00 – 16:00 for one week	May. July. September 2014	[14]	Two Closed dynamic chamber LI-COR 6400	0.52	9	768	85.3
Observational	Forest	USA, Florida	Moderately seasonal	Transect	Two sampling dates	Semi continuous	October 1995 and January 1996	[15]	Closed dynamic chamber LI-COR 6252	0.55	0.0625	12	192
Observational	Forest	USA. California	Mediterranean	Grid-based	Once or twice per month	6-10 measurements per day, from early morning to late	Summer fall and early winter of 1998	[16]	Closed dynamic chamber LI-COR 6400-09	0.60	0.18	18	100

						afternoo n							
Observational	Forest	Italy	Mediterr anean	Rando m	14 sampling dates	In the morning	Oct 2000 to Aug 2002	[17]	Closed dynamic chamber EGM-2 PP-Systems	0.63	1	100	100
Observational	Forest	France	Tempera te	Grid-based		All day long	Two times in June of 2003 and July 2004	[1]	Closed dynamic chamber LI-COR 6252	0.26	0.42	42	100
Observational	Forest	Israel	Mediterr anean semiarid	Rando m	Once per day	Between midday and early afternoo n	Octobe r 2000-Septem ber 2006	[18]	Closed dynamic chamber LI-COR 6400	0.17	0.27	29	107
Observational	Tree plantat ion	French Guyana	Tropical. wet	Rando m	Five sampling dates	10:00 – 16:00	Februar y to Octobe r 2005	[19]	Closed dynamic chamber EGM-4 PP-Systems	0.63	0.044	6	136
Observational	***Forest***	***Central Italy***	***Mediterr ranean***	***Rando m***	***Sixteen sampling dates***	***11:00 – 16:00***	***Februar y 2009 to***		***Closed dynamic chamber EGM-1***	***0.64***	***50***	***50***	***150***

							January 2009		***PP-Systems***				
Observational	Forest	Malaysia	Tropical	Grid-based	In each sampling occasion. measurements were done every one to four days: 20 times over 22 months (500 measurements in total)		between one and five month intervals during May 2002 to February 2004	[20]	Closed dynamic chamber LI-COR 6400-09	0.84	0.16	25	156.25
Observational	Forest	Germany	Suboceanic subcontinental	Transect-based	Every 2 to 6 weeks for a total of 27 measurement campaigns	During the daytime	July 2000 to July 2001	[13]	Closed dynamic chamber LI-COR 6400-09	0.49	0.2078	36	173

Observational	Abandoned field	Spain	Mediterranean	Random	Monthly	9:00 – 12:00	January 2006 and December 2007	[4]	Closed dynamic chamber LI-COR 6400-09	0.30	0.15	30	200
Observational	Young tree plantation	Japan	Asian monsoon	Grid-based	Four dates: in May, July, September, and November	From 11:00 to 13:00	2005	[21]	Closed dynamic chamber LI-COR 6400-09	0.41	0.0625	16	256
Observational	Forest	Germany	Suboceanic subcontinental	Grid-based	Three measurement campaigns	During the daytime of two or three days each campaign.	May. June. and July 2001	[13]	Closed dynamic chamber LI-COR 6400-09	0.49	0.5	144	288
Observational	Forest	USA. Massachusetts	Cool. moist temperate	Random	Once per week	Between 18 June and 21 August	2002	[22]	Closed dynamic chamber LI-COR 6252	0.20	0.04	12	300
Observational	Forest	Italy	Alpine	Random	Irregularly. for a total of		spring 2000 to	[8]	Closed dynamic chamber		0.07	24	343

					14 sampling dates		spring 2002		LI-COR 6400				
Observational	Forest	USA. Georgia	Humid subtropical	Grid-based	Six sampling dates	Morning, midday, afternoon	July to August 2012	23	Closed dynamic chamber LI-COR 6400	0.60	0.0625	25	400
Observational	Forest	North eastern China	Continental monsoon	Random	Every other week. avoiding rain	10:00 – 16:00	From May to October 2013	24	Closed dynamic chamber LI-COR 6400	0.44	0.03	18	600
Observational	Savannah	USA. California	Mediterranean	Transect-based	3–4 times per day	?	July 2001 December 2003.	25	Closed dynamic chamber LI-COR 6400-09	0.20	0.0156	11	705
Designed	Forest-steppe mosaic	Hungary	Transition between Atlantic temperate and continental	Random	Once per month, since April to November	Twice a day. between 2:30 and 7:30 and between 11:30 and 16:30	2003 to 2009	26	ADC Leaf Chamber Analyzer 4 and PLC & 2250 Soil hood. ADC BioScientific. Hoddesdon**	0.13	0.006	9	1500

Designed	Shrubland	Italy	Mediterranean semiarid	Random	Once per month	Twice a day. at sunrise and between 12:00 and 14:00	2002 to 2004	[27]	Closed dynamic chamber EGM-3 PP Systems	0.42	0.0075	15	2000
Observational	Forest	Spain	Mediterranean	Grid-based	Two consecutive days over two consecutive weeks in spring	Twice a day	2010	[28]	Both closed dynamic chamber EGM-4 PP-Systems and soda lime**	0.43	0.052	195	3750
Observational	Forest	France	Temperate	Transect-based	?	All day long	Two times in June of 2003 and July 2004	[1]	Closed dynamic chamber LI-COR 6252	0.26	0.0022	28	12727

Notes: *In case of transect-based sampling design the monitored surface was inferred by assuming the sum of the areas of the tangent circles centred on the sampled spots. **No collars inserted in soil were used for measurements

Table S2. Pedoclimatic data at each sampling date.

Date	min	mean	max	Litter	Soil	Dummy variable	SD per stratum	mean	sd	min	max
	°C			RH %				g CO_2 m^{-2} h^{-1}			
02-25-2008	8.2	9.9	11.6	17.45	34.84	Dry	1.000	0.46	0.22	0.13	0.97
03-12-2008	9.7	10.4	11.2	28.64	12.39	Moist	0.982	0.66	0.21	0.27	1.21
04-01-2008	11.0	12.9	16.2	53.18	18.46	Moist	1.250	0.66	0.42	0.11	2.33
04-14-2008	11.2	12.4	14.5	59.08	17.50	Moist	1.234	0.77	0.35	0.08	1.91
04-30-2008	12.6	13.7	16.3	43.71	13.69	Moist	1.908	1.04	0.56	0.31	3.58
05-12-2008	14.4	16.1	19.2	15.42	6.72	Dry	0.727	0.45	0.24	0.01	1.15
05-23-2008	14.7	16.2	18.6	40.53	6.53	Moist	1.469	1.07	0.44	0.54	2.40
06-09-2008	16.7	18.3	22.6	49.39	4.60	Moist	2.167	1.37	0.42	0.75	2.35
06-30-2008	21.9	24.2	29.3	17.45	4.27	Moist	1.028	0.66	0.27	0.22	1.40
07-23-2008	18.5	21.1	24.6	10.98	4.69	Dry	1.501	0.42	0.31	0.01	1.05
08-29-2008	20.3	23.4	27.5	10.72	3.34	Dry	0.854	0.20	0.14	0.01	0.57
09-18-2008	16.7	18.4	22.1	39.34	8.10	Moist	1.757	0.81	0.31	0.33	1.69
10-14-2008	16.1	16.9	19.4	14.27	4.03	Dry	0.467	0.19	0.11	0.01	0.48
11-10-2008	11.7	13.9	15.4	35.50	4.43	Moist	0.868	0.73	0.22	0.40	1.28
12-17-2008	7.8	9.3	10.6	66.24	12.17	Moist	0.742	0.41	0.21	0.11	1.19
01-17-2009	6.2	7.5	8.6	46.80	11.83	Moist	1.189	0.33	0.15	0.12	0.85

.

Table S3. Comparison of three marginal mixed effects models, where soil temperature was considered to be in linear. quadratic or cubic relationship with R_s The CO_2 flux was considered not transformed, but when log- or square root-transformed the results were similar

Soil temperature	Model	df	BIC	logLik	Test	L.Ratio	p-value
linear	1	23	203.993	-25.4000			
quadratic	2	25	205.928	-19.7069	1vs2	11.38620	0.0034
cubic	3	27	217.793	-18.9786	2vs3	1.45661	0.4827

Table S4. Comparison of two mixed effects models. One with soil water content and the other with litter water content, both as continuous variable. The CO_2 flux was considered not transformed, but when log- or square root-transformed the results were similar.

	Model	df	BIC	logLik
Litter_RH quadratic	1	25	205.928	-19.7069
soil_RH quadratic	2	25	454.170	-143.8278

Table S5. Characteristics of six models as resulting from the combination of the CO_2 flux (as such, log- or sqrt transformed) with the two variables (litter moisture as continuous or dummy variable)

Model	df	BIC	logLik
flux ~ LitterRH + …	25	205.928	-19.707
flux ~DummyRH + …	25	225.766	-29.626
log(flux) ~ LitterRH + …	25	999.956	-416.721
log(flux) ~ DummyRH + …	25	1029.745	-431.615
sqrt(flux) ~ LitterRH + …	25	-560.912	363.713
sqrt(flux) ~ DummyRH + …	25	-545.998	356.256

Figures

Figure S1. Quantile-quantile plots of residuals obtained from six models described in the main text, whose characteristics are reported in Table 3 SI. Non transformed data shows the largest departure form normality, particularly in the two tails of the distribution.

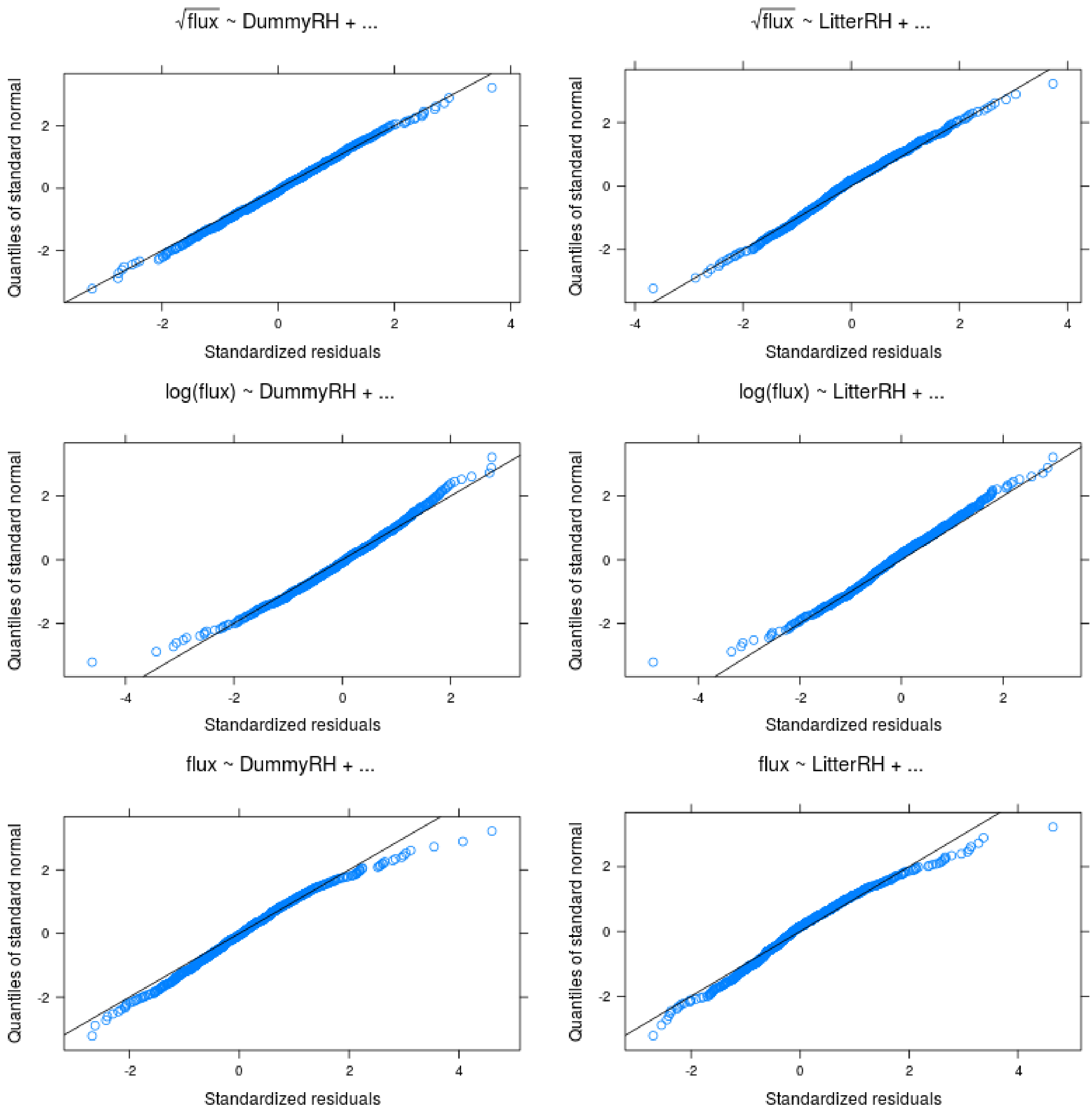

Figure S2. The pine-dominated forest under study seen from the inside (photo by G.Certini).

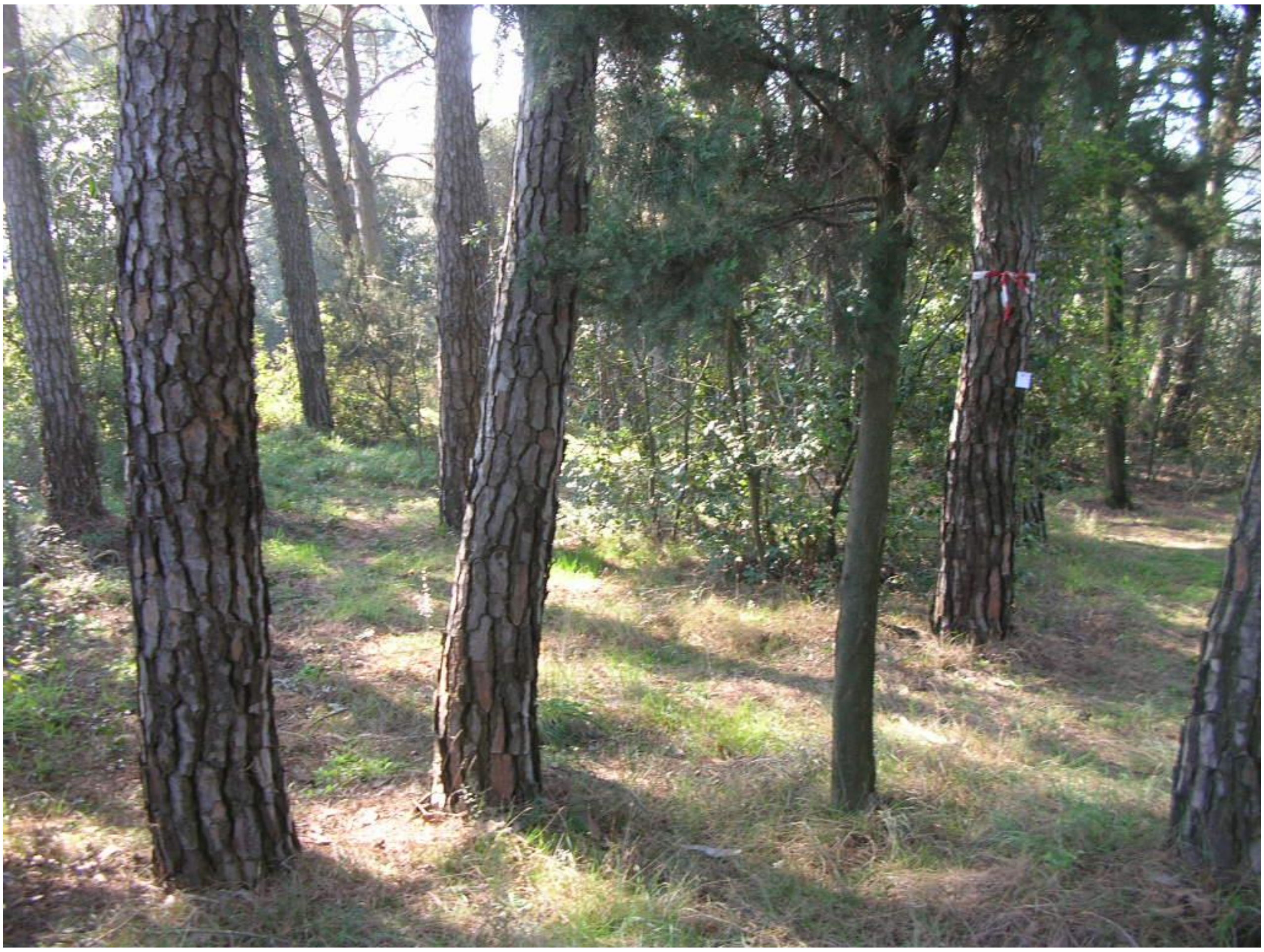

Figure S3. The mixed-effects model [1] described in the text. The number in the strip of each panel is the sampled spot, while Dry and Moist refer to a dummy variable describing the soil conditions on sampling dates. The dashed lines indicate the random part of the model while the solid ones indicate the fixed part.

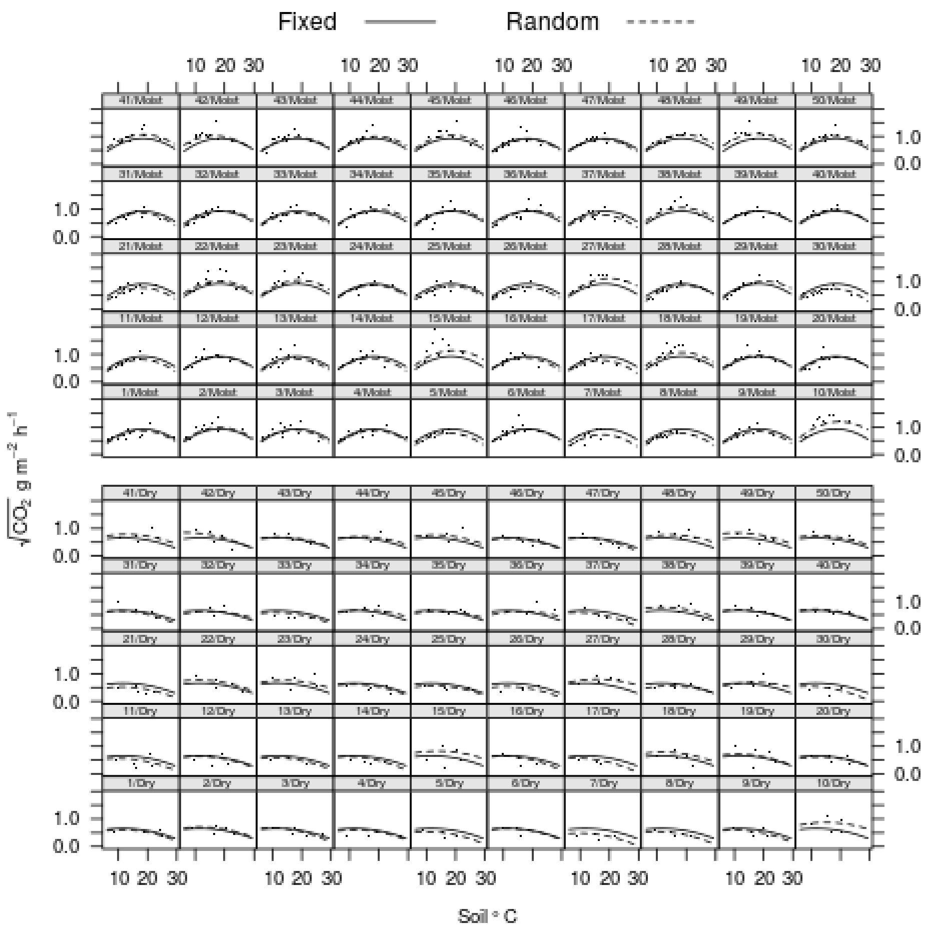

Figure S4. The residuals of the mixed-effects model [1] described in the text. No apparent deviations from normality are visible

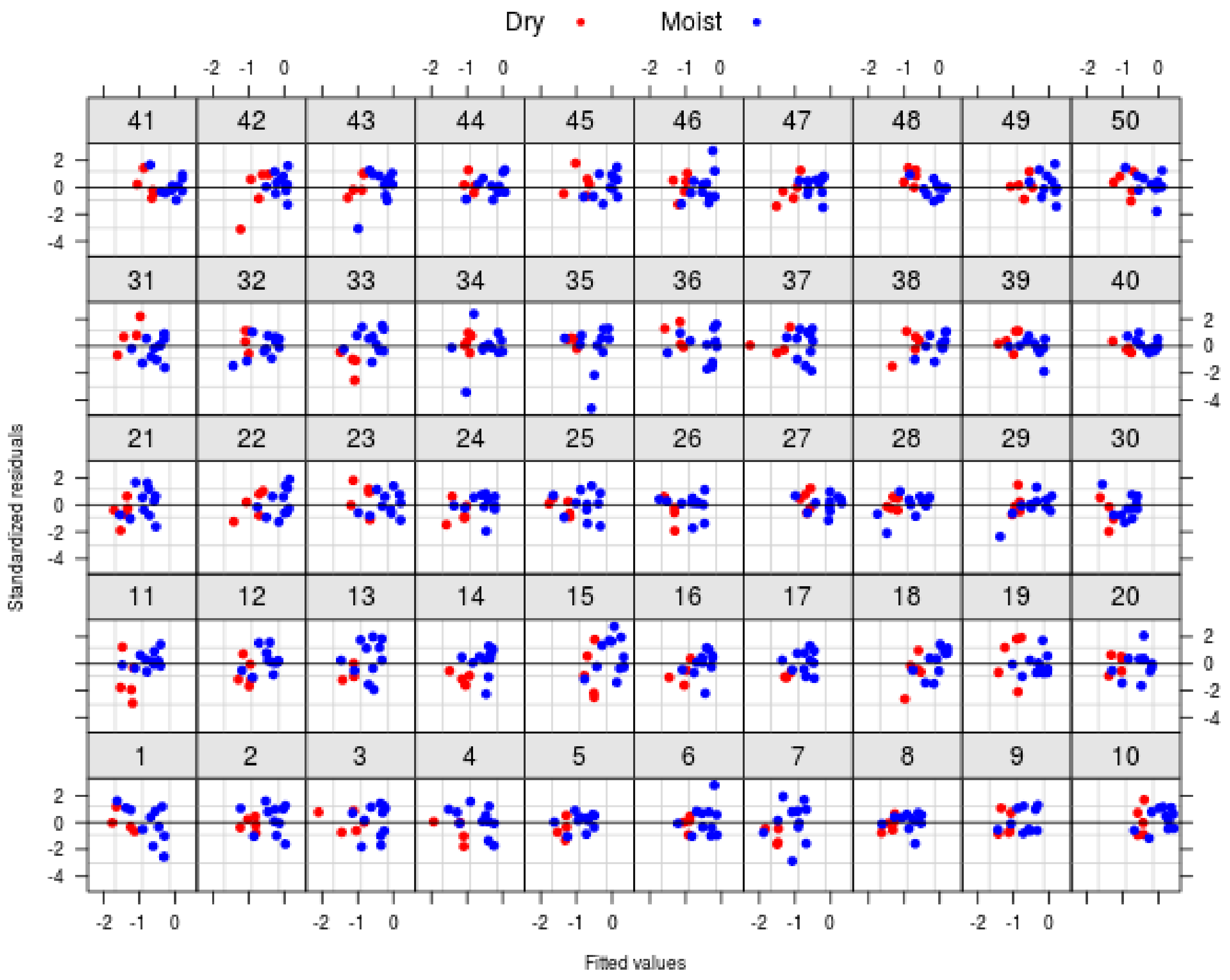

References

1. Ngao, J. *et al.* Spatial variability of soil CO_2 efflux linked to soil parameters and ecosystem characteristics in a temperate beech forest. *Agricultural and Forest Meteorology* **154**, 136–146 (2012).
2. Emran, M., Gispert, M. & Pardini, G. Comparing measurements methods of carbon dioxide fluxes in a soil sequence under land use and cover change in North Eastern Spain. *Geoderma* **170**, 176–185 (2012).
3. Dore, S., Fry, D. L. & Stephens, S. L. Spatial heterogeneity of soil CO2 efflux after harvest and prescribed fire in a California mixed conifer forest. *Forest Ecology and Management* **319**, 150–160 (2014).
4. Almagro, M., López, J., Querejeta, J. & Martínez-Mena, M. Temperature dependence of soil CO_2 efflux is strongly modulated by seasonal patterns of moisture availability in a Mediterranean ecosystem. *Soil Biology & Biochemistry* **41**, 594–605 (2009).
5. Fan, Z., Neff, J. C. & Hanan, N. P. Modeling pulsed soil respiration in an African savanna ecosystem. *Agricultural and Forest Meteorology* **200**, 282–292 (2015).
6. Adachi, M. *et al.* Required sample size for estimating soil respiration rates in large areas of two tropical forests and of two types of plantation in Malaysia. *Forest Ecology and Management* **210**, 455–459 (2005).
7. Adachi, M., Ishida, A., Bunyavejchewin, S., Okuda, T. & Koizumi, H. Spatial and temporal variation in soil respiration in a seasonally dry tropical forest, Thailand. *Journal of Tropical Ecology* **25**, 531–539 (2009).
8. Rodeghiero, M. & Cescatti, A. Spatial variability and optimal sampling strategy of soil respiration. *Forest Ecology and Management* **255**, 106–112 (2008).

9. Yuste, J. C., Janssens, I. A. & Ceulemans, R. Calibration and validation of an empirical approach to model soil CO_2 efflux in a deciduous forest. *Biogeochemistry* **73**, 209–230 (2005).
10. Zhang, L., Chen, Y., Zhao, R. & Li, W. Significance of temperature and soil water content on soil respiration in three desert ecosystems in Northwest China. *Journal of Arid Environments* **74**, 1200–1211 (2010).
11. Fernandez, D., Neff, J., Belnap, J. & Reynolds, R. Soil Respiration in the Cold Desert Environment of the Colorado Plateau (USA): Abiotic Regulators and Thresholds. *Biogeochemistry* **78**, 247–265 (2006).
12. Subke, J.-A., Reichstein, M. & Tenhunen, J. D. Explaining temporal variation in soil CO_2 efflux in a mature spruce forest in Southern Germany. *Soil Biology and Biochemistry* **35**, 1467–1483 (2003).
13. Søe, A. R. B. & Buchmann, N. Spatial and Temporal Variations in Soil Respiration in Relation to Stand Structure and Soil Parameters in an Unmanaged Beech Forest. *Tree physiology* **25**, 1427–36 (2005).
14. Shi, B., Gao, W., Cai, H. & Jin, G. Spatial variation of soil respiration is linked to the forest structure and soil parameters in an old-growth mixed broadleaved-Korean pine forest in northeastern China. *Plant Soil* **400**, 263–274 (2016).
15. Fang, C., Moncrieff, J. B., Gholz, H. L. & Clark, K. L. Soil CO2 efflux and its spatial variation in a Florida slash pine plantation. *Plant and Soil* **205**, 135–146 (1998).
16. Xu, M. & Qi, Y. Soil-surface CO2 efflux and its spatial and temporal variations in a young ponderosa pine plantation in northern California. *Global Change Biology* **7**, 667–677 (2001).
17. Tedeschi, V. *et al.* Soil respiration in a Mediterranean oak forest at different developmental stages after coppicing. *Global Change Biology* **12**, 110–121 (2006).
18. Grünzweig, J. M. *et al.* Water limitation to soil CO_2 efflux in a pine forest at the semiarid “timberline”. *J. Geophys. Res.* **114**, G03008 (2009).

19. Bréchet, L. *et al.* Do tree species characteristics influence soil respiration in tropical forests? A test based on 16 tree species planted in monospecific plots. *Plant and Soil* **319**, 235–246 (2009).
20. Ohashi, M., Kume, T., Yamane, S. & Suzuki, M. Hot spots of soil respiration in an Asian tropical rainforest. *Geophys. Res. Lett.* **34**, L08705 (2007).
21. Lee, N.-Y. & Koizumi, H. Estimation of the number of sampling points required for the determination of soil CO_2. Efflux in two types of plantation in a temperate region. *Journal of Ecology and Field Biology* **32**, 67–73 (2009).
22. Savage, K. E. & Davidson, E. A. A comparison of manual and automated systems for soil CO_2 flux measurements: trade-offs between spatial and temporal resolution. *J Exp Bot* **54**, 891–899 (2003).
23. ArchMiller, A. A., Samuelson, L. J. & Li, Y. Spatial variability of soil respiration in a 64-year-old longleaf pine forest. *Plant Soil* **403**, 419–435 (2016).
24. Shi, B. & Jin, G. Variability of soil respiration at different spatial scales in temperate forests. *Biol Fertil Soils* **52**, 561–571 (2016).
25. Tang, J. & Baldocchi, D. D. Spatial–temporal variation in soil respiration in an oak–grass savanna ecosystem in California and its partitioning into autotrophic and heterotrophic components. *Biogeochemistry* **73**, 183–207 (2005).
26. Lellei-Kovács, E. *et al.* Thresholds and interactive effects of soil moisture on the temperature response of soil respiration. *European Journal of Soil Biology* **47**, 247–255 (2011).
27. de Dato, G., De Angelis, P., Sirca, C. & Beier, C. Impact of drought and increasing temperatures on soil CO_2 emissions in a Mediterranean shrubland gariga. *Plant and Soil* **327**, 153–166 (2010).
28. Barba, J., Yuste, J. C., Martìnez-Vilalta, J. & Lloret, F. Drought-induced tree species replacement is reflected in the spatial variability of soil respiration in a mixed Mediterranean forest. *Forest Ecology and Management* **306**, 79–87 (2013).
